# High-Throughput Screening for a Moderately Halophilic Phenol-Degrading Strain and Its Salt Tolerance Response

**DOI:** 10.3390/ijms160611834

**Published:** 2015-05-25

**Authors:** Zhi-Yan Lu, Xiao-Jue Guo, Hui Li, Zhong-Zi Huang, Kuang-Fei Lin, Yong-Di Liu

**Affiliations:** 1 State Environmental Protection Key Laboratory of Environmental Risk Assessment and Control on Chemical Process, School of Resources and Environmental Engineering, East China University of Science and Technology, Shanghai 200237, China; E-Mails: lzy1009a@163.com (Z.-Y.L.); foreverkimi1017@126.com (X.-J.G.); z2cyj@163.com (Z.-Z.H.); kflin@ecust.edu.cn (K.-F.L.); 2 School of Chemical Engineering, Shanghai University of Engineering Science, Shanghai 201620, China

**Keywords:** high-throughput screening, moderately halophilic bacteria, phenol-degrading, salt tolerance

## Abstract

A high-throughput screening system for moderately halophilic phenol-degrading bacteria from various habitats was developed to replace the conventional strain screening owing to its high efficiency. Bacterial enrichments were cultivated in 48 deep well microplates instead of shake flasks or tubes. Measurement of phenol concentrations was performed in 96-well microplates instead of using the conventional spectrophotometric method or high-performance liquid chromatography (HPLC). The high-throughput screening system was used to cultivate forty-three bacterial enrichments and gained a halophilic bacterial community E3 with the best phenol-degrading capability. *Halomonas* sp. strain 4-5 was isolated from the E3 community. Strain 4-5 was able to degrade more than 94% of the phenol (500 mg·L^−1^ starting concentration) over a range of 3%–10% NaCl. Additionally, the strain accumulated the compatible solute, ectoine, with increasing salt concentrations. PCR detection of the functional genes suggested that the largest subunit of multicomponent phenol hydroxylase (*LmPH*) and catechol 1,2-dioxygenase (*C12O*) were active in the phenol degradation process.

## 1. Introduction

Phenol and phenolic compounds are hazardous pollutants in the environment discharged from a variety of industries, including petroleum coking, pharmaceuticals, chemicals, printing, dyeing, pesticides, coal processing, *etc.* [1]. Because of their toxicity, removal of phenols from industrial wastewater effluent before their discharge into receiving water bodies is thus obligatory [2]. Biological treatment could remove phenol efficiently [3,4]; however, it often faces great challenges due to the high salinity, which inhibits the growth of activated sludge cultures. Screening for salt tolerance and phenol-degrading strains was thus important for improving the performance of biological treatment. Such halophilic bacteria isolated from saline environment would be helpful to remove phenol from high-salinity industrial wastewater [5]. In particular, moderately halophilic bacteria have been considered the most versatile group with great potential for phenol biodegradation, which can grow over a wide range of salinity. Recently, many moderately halophilic bacteria have been isolated from different saline habitats [6,7,8], which had good prospects for the treatment of high-salinity organic wastewater. However, the conventional strain screening was too tedious to obtain enough strains, and the detailed information about their salt tolerance and degradation mechanism was limited.

Conventional strain screening typically involves plate-spreading and repeated streaking, which makes the screening procedure very time-consuming, inefficient and limited to a few environmental samples. Therefore, a new screening strategy is required to improve the efficiency. High-throughput screening technology is a new technique widely used in strain breeding, mutation analysis, drug testing, *etc.* [9,10,11]. However, its application to the screening for degrading strains from saline habitats has not been reported. An integrated high-throughput screening strategy includes high-throughput cultivation and the matched high-throughput analysis. To solve the low-throughput problem, firstly, a deep well microplate was used for cultivation of enrichments instead of shake flasks or tubes [10]. Secondly, measurement of phenol concentrations was performed in microplates instead of using the conventional spectrophotometric method or HPLC. Thus, high-throughput screening was achieved through micro-scale cultivation combined with the micro-quantity test.

In a previous study, many moderately halophilic bacteria have been reported to degrade phenol as a carbon and energy source [12]. The compatible solutes played an important role in moderately halophilic bacteria maintaining osmotic balance between the cytoplasm and the external salt environment. They were highly water-soluble, low molecular weight substances, including sugars, alcohols, amino acids, betaine, ectoine and its derivatives [13]. Among these, ectoine was the most common compatible solute. In addition, PCR detection of the genotypes expressed during the bacterial phenol-degrading process has been used to determine the presence of the phenol-biodegrading pathway. The initial conversion step of phenol involved in aerobic phenol biodegradation was carried out by phenol hydroxylase on the central intermediate, catechol. Then, catechol was further degraded via the ortho- or meta-cleavage pathway responsible for catechol 1,2-dioxygenase (*C12O*) or catechol 2,3-dioxygenase (*C23O*).

This study developed a high-throughput screening system for moderately halophilic phenol-degrading bacteria. The enrichments were cultivated in 48 deep well microplates, and phenol was analyzed by a photometric test performed in microplates. The high-throughput screening system was firstly applied to cultivate halophilic phenol-degrading enrichments from the saline habitats under different salt concentrations. Additionally, a phenol-degrading strain was isolated and characterized in terms of phenol-degrading ability at different salinities. Furthermore, this study revealed the salt tolerance response and detected several phenol-degrading genes of the isolated strain.

## 2. Results and Discussion

### 2.1. High-Throughput Cultivation of Halophilic Bacterial Communities

**Figure 1:**
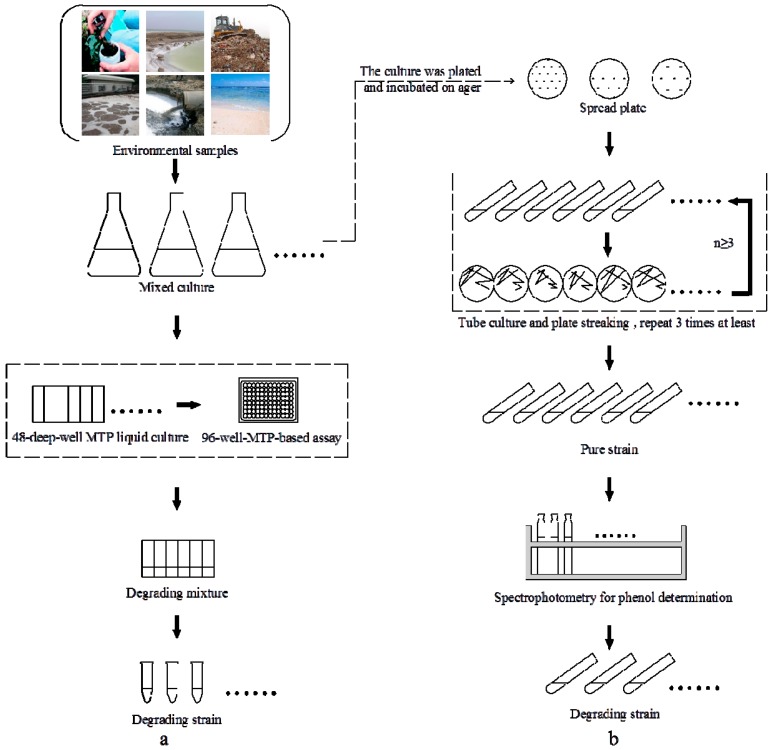
An integrated procedure for high-throughput screening. (**a**) High-throughput screening for the degrading strain; (**b**) Traditional method for the degrading strain.

The halophilic bacterial communities were cultivated in 48 deep well microplates under different NaCl concentrations. The high-throughput screening procedure (Figure 1) was designed based on two characteristics, salt tolerance and phenol degradation, for environmental samples. Using this process, we screened halophilic bacterial communities from 43 bacterial enrichments and harvested a bacterial community that could degrade phenol efficiently in a broad range of salinities. Previous studies have reported halophilic or halotolerant microorganisms from salt environments using conventional screening techniques. For example, Leitão *et al.* [14] isolated a halotolerant strain of *Penicillium chrysogenum* from a salt mine in Portugal that could degrade at least 300 mg·L^−1^ phenol. Arulazhagan *et al.* [15] enriched a halotolerant bacterial consortium from mixed saline water samples collected from India. Haddadi and Shavandi [16] isolated a moderately halophilic strain of *Halomonas* sp. strain PH2-2 from petroleum-contaminated soil in Iran that could degrade 400 mg·L^−1^ phenol with a removal efficiency of 95% at 7% NaCl. However, these conventional screenings were tedious, had a large material requirement and low-throughput, which were applied to limited samples. This high-throughput screening system processed 43 samples for phenol degradation experiments targeting six salinity levels, and only six microplates were needed to replace 258 flasks or tubes, which greatly reduced the volume occupied by the shaker and the reagent consumption. Compared with shake flasks or tubes handling samples one by one, deep well microplates were intensive in cultivating samples simultaneously. Therefore, the screening was condensed and performed with small sample quantities. It could be used for the mass cultivation of phenol-degrading enrichments from various saline habitats.

### 2.2. High-Throughput Phenol Measurement Performed in Microplates

**Figure 2:**
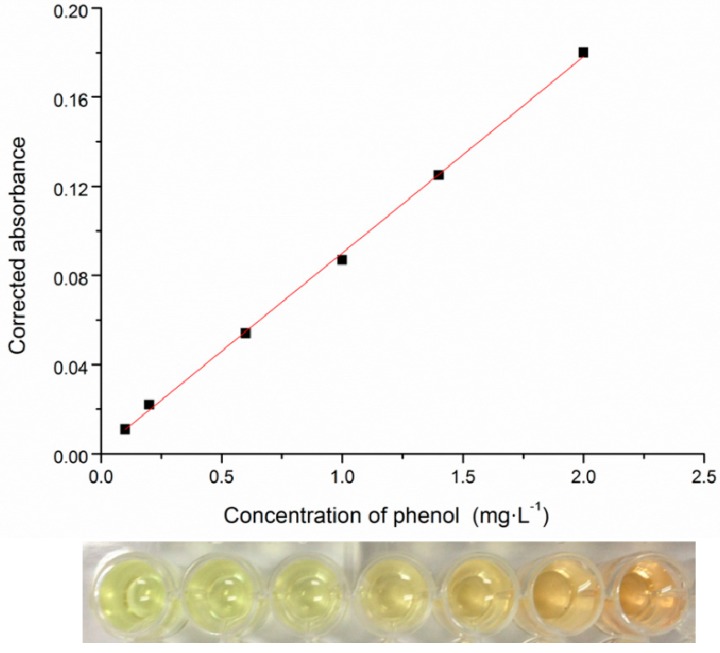
The correlation between phenol concentration and the absorbance of the generated indoxyl antipyrine in a 96-well microplate screening system (**upper** panel); the color formation of indoxyl antipyrine at different phenol concentrations (**lower** panel).

This study established a high-throughput assay for phenol measurement performed in 96-well microplates based on the 4-aminoantipyrine color reaction principle, which resolved the low throughput bottleneck in conventional measurement for phenol concentrations. This assay had a good linear detection range of phenol from 0.1–2 mg·L^−1^. The standard curve was *y* = 0.08808*x* + 0.00203 (*R*^2^ = 0.9988) (Figure 2), revealing that the assay was reliable. The relative standard deviation (RSD) between wells was less than 3%, indicating no significant difference between them. The data based on the high-throughput assay were in close agreement with those obtained from the conventional spectrophotometric method (ASTM Standard D1783-01,2012) and HPLC [17]; the high correlation coefficient (0.976 and 0.965) were obtained between them by statistical analysis, revealing the accuracy of the assay. Therefore, it could be used for phenol determination in a high-throughput screening system. The conventional spectrophotometric method was widely used for the measurement of phenol. However, it was time-consuming, with a large chemical reagent requirement, and inefficient, which was impractical for a high-throughput system. The coloring reaction was carried out in the colorimetric tubes. Each sample was sequentially detected, which resulted in detection errors. HPLC was a sensitive and fast method. However, the samples are required to flow through the column for separation in sequence, resulting in a low detection throughput. This study establishes a high-throughput phenol determination method based on conventional colorimetric principles. Colorimetric tubes were replaced with 96-well microplates, and the spectrophotometer was replaced with the microplate spectrophotometer, which contributed to the micro-volume quantification. The microplate spectrophotometer was able to read the information of the absorbance for the entire plate and to detect 96 samples simultaneously, achieving the purpose of high-throughput detection. The eight-channel pipettes transferred bacterial culture to 96-well microplates in 40 s; the absorbance values of all of the wells were obtained in 6 s using the microplate reader; data could be obtained well within 1 h. Each test had a 96-throughput. In this study, only three tests were required to obtain 258 phenol concentrations.

**Figure 3:**
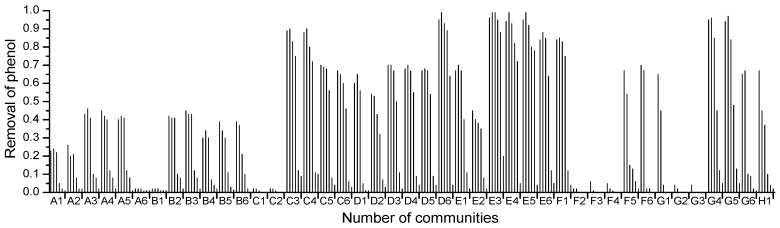
Results of high-throughput screening for halophilic bacterial phenol-degrading communities.

**Figure 4:**
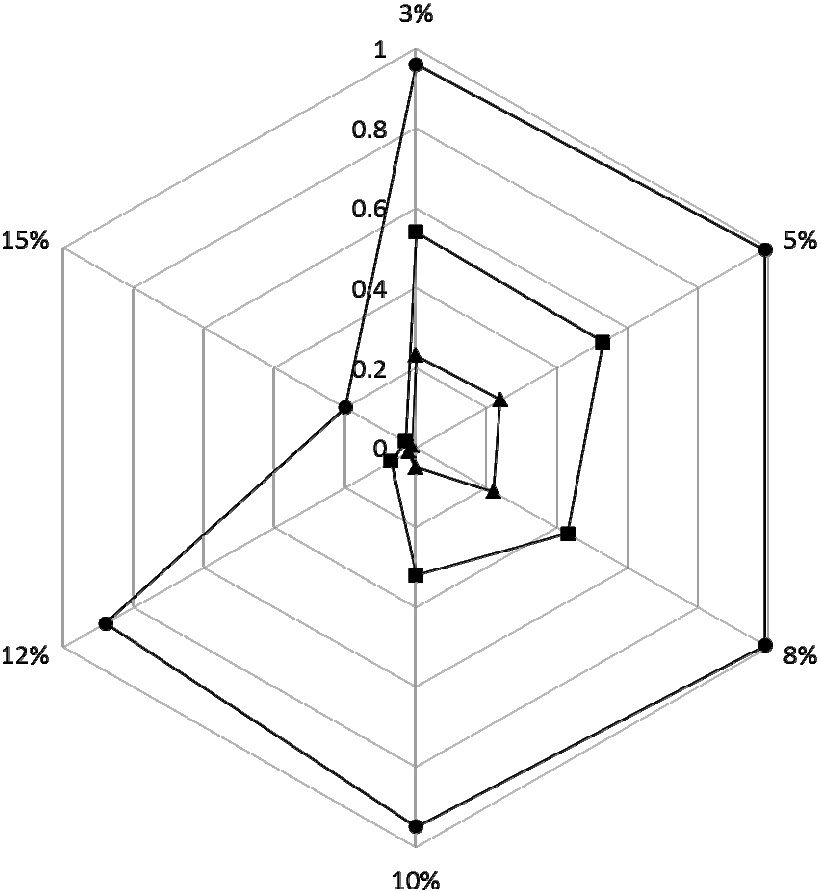
Biodegradation of phenol by halophilic bacterial communities at 3%, 5%, 8%, 10%, 12%, 15% NaCl (*w*/*v*); ●: E3, ■: D2, ▲: A1.

The ability to degrade phenol by 43 halophilic bacterial communities (No. A1–H1) cultivated in 48 deep well microplates was investigated. The residual phenol concentration in the 48 deep well microplates was measured by the high-throughput phenol measurement. The results showed that, of the 43 halophilic bacterial communities enriched, 10 degraded phenol above 50% in the presence of 3%–10% (*w*/*v*) NaCl (Figure 3). They were C3, C4, D6, E3, E4, E5, E6, F1, G4 and G5, which came from Daqing saline-alkaline soil, Shanghai Old Port Landfill Factory waste and the Shanghai Sinopec Gaoqiao petrochemical factory. By comparing with the phenol degradation of three bacterial communities (E3, D2, A1) under various salt conditions, the results showed that the phenol removal efficiency was higher under low salt conditions than the high salt conditions (Figure 4). When the salinity reached 12%, the phenol removal was severely inhibited by the salinity. The community E3 had the best degradation ability over a wide range of salinities, which came from Shanghai Old Port Landfill Factory waste. Five pure strains were then isolated from the E3 community. Among them, strain 4-5 showed optimal phenol-degrading characteristics.

### 2.3. 16S rRNA Gene Sequence Analysis and Identification of the Isolated Strain

**Figure 5:**
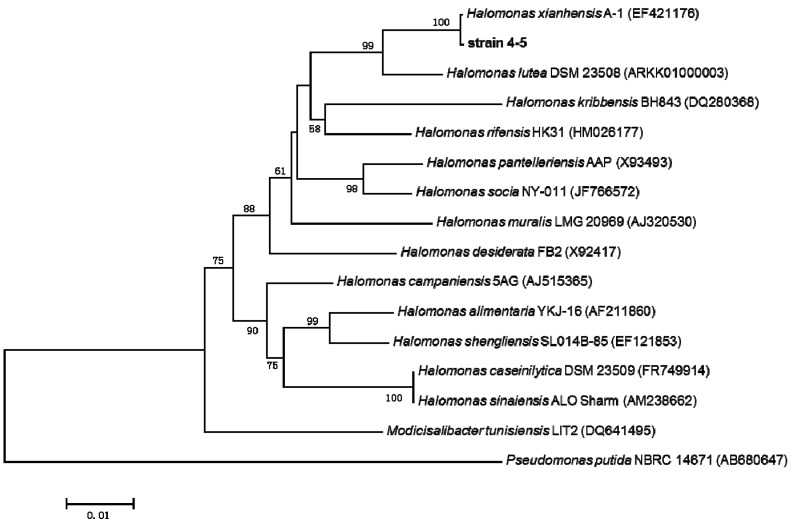
Phylogenetic tree based on 16S rRNA sequences, constructed by the neighbor-joining method, showing the position of strain 4-5 and representatives of some related strains. *Pseudomonas putida* NBRC 14671 was used as an out group. Bootstrap values were 1000 replicates, in which ≥50% were reported near the corresponding nodes. The scale bar indicates the percentage of genetic distance.

Strain 4-5 with optimal phenol-degrading characteristics was identified as a member of the *Halomonas* genus using 16S rDNA sequence analysis. Phylogenetic relationships between *Halomonas* sp. strain 4-5 and other species of the *Halomonas* genus were constructed on the basis of their 16S rRNA gene sequences (Figure 5). The closest relative of strain 4-5 was *Halomonas xianhensis* A-1 with a 16S rRNA sequence similarity of 99%. Phylogenetic analysis using the neighbor-joining algorithm with *Pseudomonas putida* NBRC 14671 as the out group also proved that the strain belonged to the *Halomonas* genus. The genus *Halomonas* was one of the largest among the moderately halophilic bacteria, which was originally proposed by Vreeland and primarily isolated from hypersaline or saline environments [18,19]. Members of the *Halomonas* genus were typically moderately halophilic bacteria, which contributed to the treatment of high-salinity industrial wastewater. *H. xianhensis* sp. nov. was first isolated by Zhao *et al.* [20] from the crude oil-contaminated soil in Shengli oilfield. In this study, the same species was isolated from the mineralized waste, and further studies of its salt tolerance response and degradation pathway were conducted. 

### 2.4. Phenol Degradation by Halomonas sp. Strain 4-5 under Various Salinities

**Figure 6:**
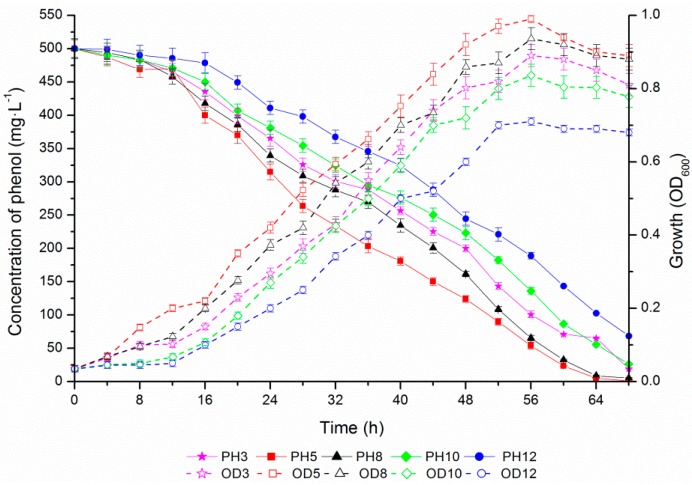
Biodegradation of phenol by *Halomonas* sp. strain 4-5 at different salt concentrations with 500 mg·L^−1^ phenol. The experiments were carried out in 125-mL serum bottles containing 50 mL of mineral salts medium (MSM) with 5% inoculation. PH stands for phenol concentration, and OD stands for optical density. Numbers correspond to NaCl concentration. Data are the mean of triplicate bottles, and bars indicate ± the standard deviation.

The effects of salinity on the growth and phenol degradation of strain 4-5 was investigated in mineral salts medium (MSM) containing 500 mg·L^−1^ phenol and various concentration of NaCl (Figure 6). *Halomonas* sp. strain 4-5 showed optimal growth and phenol degradation at 5% NaCl. The strain was able to remove phenol after 68 h when cultivated in medium containing 500 mg·L^−1^ phenol and 3%, 5%, 8%, 10% and 12% NaCl. The removal rates were 96.2%, 99.8%, 98.9%, 94.7% and 86.3%, respectively. When the salinity increased to more than 12%, the phenol removal rate decreased significantly. A few studies have already reported successful phenol removal under salt conditions; most of these investigated the degradation ability in fixed salinity or with a narrow range of salinities. Peyton *et al.* [21] enriched five bacterial cultures from diverse saline environments capable of degrading phenol from 50 mg·L^−1^ to lower than 2 mg·L^−1^ at 10% (*w*/*v*) NaCl. Bonfá *et al.* [22] isolated three halophilic strains from different saline environments identified as *Halomonas organivorans*, *Arhodomonas aquaeolei* and *Modicisalibacter tunisiensis* that could grow in a medium with 10% salinity and 280 mg·L^−1^ phenol. Kobayashi *et al.* [23] separated three marine bacteria identified as *Acinetobacter* spp. EBR01, EBR02 and *C. marina* EBR04 from marine environments that could degrade 100 mg·L^−1^ phenol at 3.7% salinity. However, these studies only investigated phenol degradation under a fixed salinity. Gayathri and Vasudevan [24] examined the phenol degradation ability of a moderately halophilic bacterial consortium, which could degrade 50 mg·L^−1^ phenol with a removal rate of 95%, 99%, 93% and 89% when the NaCl concentration was 3%, 5%, 7% and 10%, respectively. By comparison, the salt tolerance range of the strain obtained in this study was up to 3%–12%. A detailed investigation of the effects of various salinities on growth and phenol degradation of the strain was reported. Additionally, the strain could degrade 500 mg·L^−1^ phenol over 94% at 3%–10% NaCl. Compared with previous reports, strain 4-5 obtained in this study had the advantage not only of a high removal rate, but also tolerance to a high salinity and initial concentration of phenol, which may contribute to phenol removal in the biological treatment of saline wastewater. Strain 4-5 was screened from the community E3, which was identified as having the best phenol-degrading capability among the 43 bacterial enrichments cultivated in the high-throughput screening system. The strain obtained from this high-throughput system had a unique phenol-degrading character compared to previous reported strains using the conventional screening techniques. The high-throughput system had the advantages not only of increasing the number of the screened samples, but also of improving the phenol-degrading ability of the gained strains.

### 2.5. Osmoprotection Response of Halomonas sp. Strain 4-5

HPLC was used to examine the ectoine accumulated in the cytoplasm of *Halomonas* sp. strain 4-5. The results showed that ectoine in cells increased as the NaCl concentration in the media increased (Table 1), indicating that the accumulation of ectoine was an important response for *Halomonas* sp. strain 4-5 to adjust the osmotic pressure when grown in high saline condition. Bursy *et al.* [25] also found out that the intracellular ectoine concentration increased as the extracellular NaCl concentration increased. Ectoine (1,4,5,6-tetrahydro-2-methyl-4-pyrimidinecarboxylic acid) was the compatible solute that most halophilic and halotolerant bacteria synthesized [26]. Ectoine was first discovered in *Ectothiorhodospira halochloris*, and a variety of halophilic bacteria was found to produce this compound [27]. In previous studies, ectoine was often reported as the main compatible solute of the strains belonging to the *Halomonas* genus [13]. *Halomonas* sp. strain 4-5 obtained in this study proved to maintain the osmotic balance by accumulating ectoine.

**Table 1:** Accumulation of ectoine in cells of *Halomonas* sp. strain 4-5 at different NaCl concentrations.

NaCl (%, *w*/*v*)	Ectoine (mg·g^−1^)
3	0.92
5	1.04
8	5.41
10	13.53
12	15.29

### 2.6. Phenol Degradation Pathway

**Figure 7:**
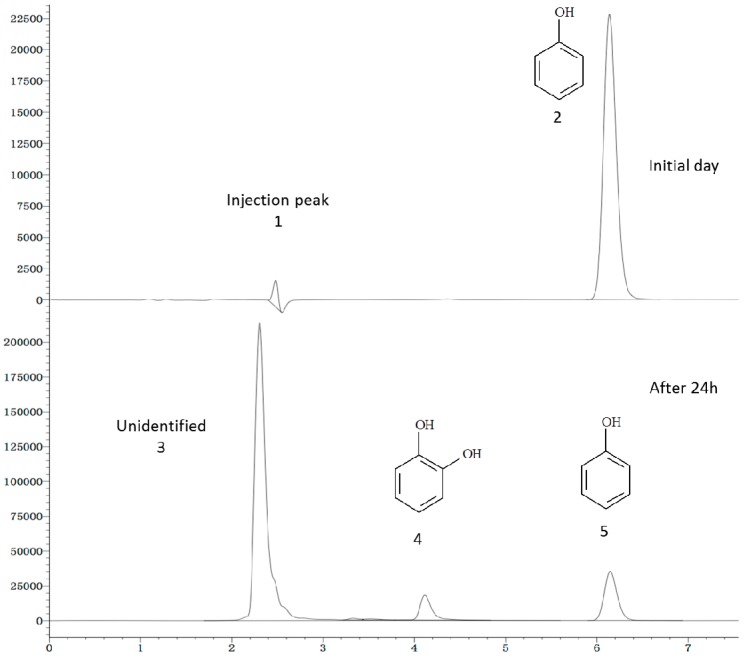
HPLC profile of phenol degradation by *Halomonas* sp. strain 4-5 at 10% (*w*/*v*) NaCl.

Studies of the phenol metabolic pathway focused mainly on the examination of the degradation intermediates and the detection of functional genes. HPLC was utilized to examine the intermediate products during the phenol degradation process. Figure 7 showed the degradation of phenol by *Halomonas* sp. strain 4-5 at 24-h time intervals. The residence times of Peak 4 and Peak 5 were 4.110 and 6.142 min, respectively. Compared with the retention time of the standard substances, Peak 4 and Peak 5 represented catechol and phenol. The HPLC profile showed that catechol was the metabolism intermediate product, indicating that degradation of phenol occurred via the catechol degradation pathway [28].

To detect the presence of the catabolic genes that encoded the key enzymes of the phenol degradation pathways, PCR amplification was performed on total DNA of *Halomonas* sp. strain 4-5 using the primers for *LmPH*, *C12O* and *C23O*. The fragments of *LmPH* and *C12O* were amplified by PCR, but PCR for *C23O* was unavailable. The BLAST analysis revealed that the *LmPH* sequences of this study exhibited 79% similarity to *Pseudomonas* sp. DHS3Y phenol hydroxylase alpha subunit gene sequences (GQ281096), which encoded the key enzyme in the first step of the biodegradation of phenol. The *C12O* sequences exhibited 83% similarity to *Halomonas organivorans* partial catA gene sequences for catechol 1,2-dioxygenase (FN997643), suggesting the presence of an ortho pathway for phenol degradation. García *et al.* [29] isolated dozens of halophilic strains of the *Halomonas* genus and found that the majority degraded phenol via the ortho pathway, which was similar to the phenol degradation pathway of *Halomonas* sp. strain 4-5 isolated in this study.

## 3. Experimental Section 

### 3.1. Sampling and Enrichment of Halophilic Bacterial Communities

For the aerobic culture, environmental samples were collected from six saline habitats, including soil and water samples (Table 2). The microcosms were prepared in 125-mL capacity serum bottles using 10 g of soil samples or 10 mL water samples and 40 mL of mineral salts medium (MSM) with 0.02% yeast extract, containing 100 mg·L^−1^ phenol. The composition of the mineral salts medium (MSM) was composed of MgCl_2_ (0.5 g·L^−1^), KH_2_PO_4_ (0.45 g·L^−1^), K_2_HPO_4_ (0.9 g·L^−1^), NH_4_Cl (0.3 g·L^−1^) and KCl (0.3 g·L^−1^). The medium was supplemented with a specified amount of added NaCl (100 g·L^−1^). The pH was adjusted to 7.0, and the medium was sterilized by autoclaving at 121 °C for 20 min. Phenol was added as a filter sterilized phenol stock solution (80 g·L^−1^) after autoclaving of the medium. The bottles were incubated at 30 °C with shaking at 150 rpm in the dark. Air in the headspace served as the source of oxygen. The enrichments were transferred 3–4 times to obtain the sediment-free cultures.

**Table 2:** Sampling description.

Sampling Site	Description	Sample Numbers
Qarhan Salt Lake	Surface soil	A1–A2
	Upper sediment	A3–A5
	Water	A6, B1
Xin Jiang Salt Lake	Surface soil	B2–B3
	Upper sediment	B4–B6
	Water	C1–C2
Saline-alkaline soil in Daqing, Heilongjiang	Surface soil	C3–C5
	Deep soil	C6, D1–D2
Shanghai Old Port Landfill Factory waste	Mineralized waste, landfill established in1990	D3–D5
	Mineralized waste, landfill established in 1991	D6, E1–E2
	Mineralized waste, landfill established in 1994	E3–E5
	Leachate-contaminated soil under the landfill pit	E6, F1
	Surface soil near the landfill site	F2
Seaside of the East China Sea	Sea water	F3–F4
	Upper sediment	F5–F6, G1
	Beach silt	G2–G3
Shanghai Sinopec Gaoqiao petrochemical factory	Biochemical reaction basin	G4–G5
	Excess sludge	G6, H1

### 3.2. High-Throughput Cultivation of Halophilic Bacterial Communities

Six 48 deep well microplates [10] were prepared with NaCl concentrations of 3%, 5%, 8%, 10%, 12% and 15% (*w*/*v*), respectively (per well: 500 mg·L^−1^ phenol, 1 mL MSM medium). Forty-three enrichments (No: A1–H1) were transferred into 48 deep well microplates and incubated 72 h at 30 °C and 200 rpm. Then, the cultures were transferred into the new 48 deep well microplates, incubated under the above conditions and transferred three times. The residual phenol concentration was tested after three transfers. Then, the optimal phenol-degrading bacterial community was screened by comparing phenol removal efficiency. Finally, pure strains were isolated from the selected bacterial community. The high-throughput screening procedure was illustrated in Figure 1.

### 3.3. Phenol Measurement Performed in 96-Well Microplates

The residual phenol concentration in the 48 deep well microplates was measured by a photometric test performed in 96-well microplates. This test was set up based on rapid condensation with 4-aminoantipyrine, followed by oxidation with potassium ferricyanide under alkaline conditions to produce the colored indoxyl antipyrine. The solution had a maximum absorption at 510 nm. Two hundred microliters of bacterial culture from 48 deep well microplates were transferred to 96-well microplates. The whole 96-well microplate was centrifuged at 2500× *g* (ZHmini-P25, ZangHan, Hangzhou, China) for 20 min. The supernatant from the bacterial culture was diluted to 300 µL and transferred to a new 96-well microplate. Three microliters of ammonia buffer solution (20%, *w*/*v*), 6 µL 4-aminoantipyrine (2%, *w*/*v*) and 6 µL potassium ferricyanide (8%, *w*/*v*) were mixed in the wells of the microplate. After 10 min, the absorbance value was monitored at 510 nm by a microplate reader (Sunrise, Tecan, Grödig, Austria). The residual phenol concentration was calculated from the absorbance value at 510 nm in accordance with the standard curve of phenol, which was plotted using the high-throughput assay with different concentrations of phenol.

### 3.4. Identification of the Isolated Strain

The total DNA from cells was extracted by a Fast DNA spin kit (ABigen, Beijing, China). The 16S rDNA gene was amplified using forward and reverse primers 27F (5ʹ-AGAGTTTGATCCTGGCTCAG-3ʹ) and 1492R (5ʹ-GGTTACCTTGTTACGACTT-3ʹ). PCR amplifications were performed in a 50-µL reaction volume that contained 1 µL of template, 2 µL of each primer, 25 µL of PCR Taqmix and 22 µL of ddH_2_O. The PCR condition included: an initial denaturation at 94 °C for 5 min, followed by 30 cycles of denaturation at 94 °C for 1 min, annealing at 54 °C for 30 s and extension at 72 °C for 1 min, with a final extension at 72 °C for 10 min. The reactions were performed on a Mastercyle Gradient thermal cycler (Eppendorf, Shanghai, China). The PCR products were purified (DNA purification kit, ABigen, Beijing, China), and the fragments were ligated into the pMD19-T vector system, according to the manufacturer’s instructions (TaKaRa, Dalian, China). After transformation with the ligation products, *E. coli* cells were grown on Luria-Bertani (LB) medium solidified with 15 g·L^−1^ agar and containing 100 µg·L^−1^ ampicillin, for 12 h at 37 °C. The white clones were verified by PCR with primers M13-47 (5ʹ-CGC CAG GGT TTT CCC AGT CAC GAC-3ʹ) and RV-M (5ʹ-GAG CGG ATA ACA ATT TCA CAC AGG-3ʹ), and those containing an insert of the correct size were sequenced (BioSune, Shanghai, China). The resulting sequences were analyzed using the BLAST software in the GenBank database of NCBI (http://www.ncbi.nlm.nih.gov). Sequence alignments and phylogenetic analyses were conducted using the Molecular Evolutionary Genetics Analysis (MEGA, Tokyo, Japan) software Version 5.05.

### 3.5. Phenol Degradation Assay

The effects of different concentrations of NaCl ranging from 3%–15% on phenol degradation of the isolated strain were examined by inoculating it into MSM containing 500 mg·L^−1^ of phenol with the above-mentioned concentrations of NaCl. Phenol and its degradation products were determined by HPLC. The cell suspensions were clarified by centrifugation at 12,000 rpm for 2 min. The culture supernatant was filtered through a 0.45-µm pore size filter, prior to analysis in HPLC (LC-20A, Shimadzu, Kyoto, Japan) equipped with a C18 column (4.6 × 150 mm, WondaSil, Shimadzu, Kyoto, Japan). The mobile phase was composed of methanol and water (50:50, *v*/*v*), and the flow rate was 0.8 mL·min ^−1^. Detection was made at 270 nm with a UV detector, and the injection volume was 20 µL. 

### 3.6. Ectoine Determination with HPLC Method

The isolated strain was maintained on MSM containing 0.4% glucose, 0.2% yeast extract and different NaCl concentration. The cells were grown until late exponential phase, harvested by centrifugation and were lyophilized; then, the dry weight of the cells was determined [30]. The cells were lysed with 400 µL of an extraction buffer (methanol/chloroform/water, 10:5:4 [*v*/*v*/*v*]) by vigorous shaking for 60 min. Equal volumes (130 μL) of chloroform and water were then added. The mixture was again shaken for 30 min and then centrifuged at 12,000 rpm for 30 min. The supernatant was recovered and dried. The pellet was resuspended in 300 μL of ammonium formate and 700 μL of acetonitrile (ACN) and was quantified by HPLC (LC-20A, Shimadzu, Kyoto, Japan) using an Amide column (4.6 × 250 mm, Inertsil, GL Sciences, Tokyo, Japan). The mobile phase consisted of ACN and ammonium formate (70:30, *v*/*v*), and the flow rate was 0.8 mL·min ^−1^. Detection was made at 210 nm with a UV detector, and the injection volume was 20 µL. The retention time of ectoine was determined by using a commercially available ectoine sample (Sigma-Aldrich, St. Louis, MO, USA).

### 3.7. Detection of Genotypes Involved in Aerobic Phenol Biodegradation

The isolated DNA of the isolated strain was screened for the presence of key phenol-degrading enzymes, including the largest subunit of multicomponent phenol hydroxylase (*LmPH*), catechol 1,2-dioxygenas gene (*C12O*) and catechol 2,3-dioxygenas (*C23O*). The genes encoding these enzymes were amplified by using the primers sets: *LmPH*f (5ʹ-CGCCAGAACCATTTATCGATC-3ʹ), *LmPH*r (5ʹ-AGGCATCAAGATCACCGACTG-3ʹ) [31]; *C12O*f (5ʹ-ACCATCGARGGYCCSCTSTAY-3ʹ), *C12O*r (5ʹ-GTTRATCTGGGTGGTSAG-3ʹ) and *C23O*f (5ʹ-GARCTSTAYGCSGAYAAGGAR-3ʹ), *C23O*r (5ʹ-RCCGCTSGGRTCGAAGAARTA-3ʹ) [29].

The PCR conditions for the amplification of *LmPH* encoding genes consisted of one cycle of initial denaturation at 94 °C for 5 min, 35 cycles of denaturation at 94 °C for 1 min, annealing at 55 °C for 35 s, extension at 72 °C for 1 min, followed by one additional cycle at 72 °C for 5 min and a final storage cycle at 4 °C. The PCR conditions for the amplification of *C12O* encoding genes consisted of an initial cycle of 5 min at 95 °C, followed by 35 cycles of: denaturation at 94 °C for 1 min, annealing at 60 °C for 1 min and extension at 72 °C for 1 min. Similar conditions were applied for the amplification of the *C23O* gene fragments, except that lower annealing temperatures were assayed (55 °C).

### 3.8. Nucleotide Sequence Accession Numbers

The GenBank accession number for the bacterial 16S rRNA gene sequence is KM894272, and those for the functional genes are KM975502–KM975503.

## 4. Conclusions

This study developed a high-throughput screening system for moderately halophilic phenol-degrading bacteria. The bacterial enrichments were cultivated from various habitats under different salt concentrations in 48 deep well microplates. Measurement of phenol concentrations was performed in 96-well microplates. Finally, a phenol-degrading strain related to *Halomonas* was isolated and characterized in terms of phenol-degrading ability at different salinities and intracellular concentrations of ectoine. Furthermore, functional genes involved in the phenol degradation process were detected. The high-throughput screening system was instructive for the rapid screening of degrading strains from the environment and contributed to the biological treatment of high-salinity phenolic wastewater.
